# M-GNN: A Graph Neural Network Framework for Lung Cancer Detection Using Metabolomics and Heterogeneous Graph Modeling

**DOI:** 10.3390/ijms26104655

**Published:** 2025-05-13

**Authors:** Maria Vaida, Jiawen Wu, Eyad Himdiat, Jean-François Haince, Rashid A. Bux, Guoyu Huang, Paramjit S. Tappia, Bram Ramjiawan, W. Rand Ford

**Affiliations:** 1 Department of Data Science, Harrisburg University of Science and Technology, Harrisburg, PA 17101, USA; jwu3@my.harrisburgu.edu (J.W.); ehimdiat@my.harrisburgu.edu (E.H.); marriotts2010@gmail.com (W.R.F.); 2 BioMark Diagnostic Solutions Inc., Quebec, QC G1P 4P5, Canada; jhaince@biomarkdiagnostics.com (J.-F.H.); ghuang@biomarkdiagnostics.com (G.H.); 3 BioMark Diagnostics Inc., Richmond, BC V6X 2W2, Canada; rahmed@biomarkdiagnostics.com; 4 Asper Clinical Research Institute and Albrechtsen Research Centre, St. Boniface Hospital, Winnipeg, MB R2H 2A6, Canada; ptappia@sbrc.ca (P.S.T.); bramjiawan@sbrc.ca (B.R.); 5 Department of Pharmacology & Therapeutics, Max Rady College of Medicine, University of Manitoba, Winnipeg, MB R3E 0T6, Canada

**Keywords:** lung cancer, metabolomics, graph neural network, heterogeneous graph

## Abstract

Lung cancer remains the leading cause of cancer-related mortality worldwide, with early detection critical for improving survival rates, yet conventional methods like CT scans often yield high false-positive rates. This study introduces M-GNN, a graph neural network framework leveraging GraphSAGE, to enhance early lung cancer detection through metabolomics. We constructed a heterogeneous graph integrating metabolomics data from 800 plasma samples (586 cases, 214 controls) with demographic features and Human Metabolome Database annotations, employing GraphSAGE and GAT layers for inductive learning on 107 metabolites, pathways, and diseases. M-GNN achieved a test accuracy of 89% and an ROC-AUC of 0.92, with rapid convergence within 400 epochs and robust performance across ten random seeds; key predictors included age, height, choline, Valine, Betaine, and Fumaric Acid, reflecting smoking and metabolic dysregulation. This framework offers a scalable, interpretable tool for precision oncology, surpassing benchmarks by capturing complex biological interactions, though limitations like synthetic data biases and computational demands suggest future validation with real-world cohorts and optimization. M-GNN advances lung cancer screening, promising improved survival through early detection and personalized strategies.

## 1. Introduction

Lung cancer remains the leading cause of cancer-related mortality globally, with projections estimating over 2 million new cases annually by 2035, driven by factors such as smoking, environmental exposures, and genetic predisposition [1]. Early detection significantly enhances survival outcomes, with the five-year survival rate for non-small cell lung cancer rising from 5% in advanced stages to nearly 60% when diagnosed at Stage I [2]. However, conventional diagnostic approaches, such as low-dose computed tomography (CT) scans and biopsies, frequently fail to detect early-stage disease, exhibiting high false-positive rates and imposing a substantial patient burden [3]. Consequently, there is an urgent need for non-invasive, precise methods to improve early detection and patient prognosis. Metabolomics, the comprehensive analysis of small-molecule metabolites in biofluids like plasma, offers a promising strategy for identifying early metabolic dysregulations linked to lung cancer, including altered amino acid and energy metabolism [4,5]. Specific metabolites, such as glycine, serine, glutamine, and lipids like sphingosine and phosphorylcholine, have emerged as potential biomarkers, reflecting tumor-driven changes in cellular proliferation and membrane synthesis [6,7]. Despite its potential, the high-dimensional and intricate nature of metabolomic data poses challenges for traditional machine learning techniques, necessitating advanced analytical tools [8]. Recent advancements in graph neural networks (GNNs) have proven effective in modeling relational data, making them ideal for capturing complex interactions within biological systems, such as those between patients, metabolites, pathways, and diseases [9,10,11,12]. GNNs have been applied to multi-omics data for cancer prognosis and subtype classification, including lung cancer [13,14,15,16,17]. However, their application in metabolomics-driven early detection remains largely unexplored, even with the enriched relational context provided by databases like the Human Metabolome Database (HMDB) [18].

This study presents M-GNN, a graph neural network framework developed for the early detection of lung cancer. The framework makes a complex graph from metabolomics data, which include 800 plasma samples (586 cases and 214 controls), combining metabolite expression levels with patient features and enhanced with HMDB annotations. GraphSAGE and Graph Attention Network (GAT) layers were utilized to enable inductive learning, aiming to improve predictive accuracy and identify significant metabolic predictors [9,19]. Building on previous metabolomics research [20,21,22,23], this approach offers a scalable and interpretable tool for precision oncology. Our work seeks to advance lung cancer screening, contributing to improved survival rates and personalized treatment strategies.

## 2. Results

**Figure 1:**
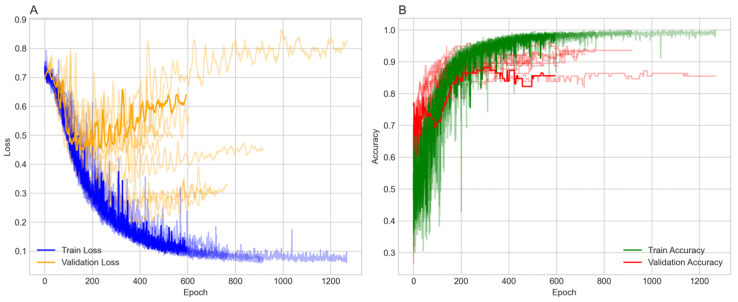
Panel (**A**) illustrates training and validation loss across epochs, while Panel (**B**) depicts training and validation accuracy over the same period.

Patient indices were split with random seeds to ensure robustness into 70% training, 15% validation, and 15% testing groups, and masks intersected with a patient mask (y≥0) to focus on labeled patient nodes only. Class imbalance was addressed in the testing and validation sets only, using the Synthetic Minority Over-Sampling Technique (SMOTE) with a sampling strategy of one and two neighbors, increasing the minority class from 214 to 586. To ensure robustness, the model was run over ten random seeds, each with a different data split. The model was trained over 1500 epochs with early stopping. The majority of the 10 runs stopped between 184 and 616 epochs and reached stable training and validation accuracies ranging from 82% to 93% (Figure 1). Figure 1A shows the training and validation losses. Both losses decrease over time, with the training loss exhibiting more variability but stabilizing around 0.3 to 0.4. The validation loss decreases more smoothly, also stabilizing in a similar range, indicating effective learning without significant overfitting in terms of loss. Figure 1B presents the training and validation accuracy, both of which increase over epochs. The training accuracy reaches approximately 90% to 95%, while the validation accuracy reaches around 0.84 to 0.95, with some fluctuations. The higher training accuracy after 400 epochs suggests a degree of overfitting, although the validation accuracy remains high.

**Figure 2:**
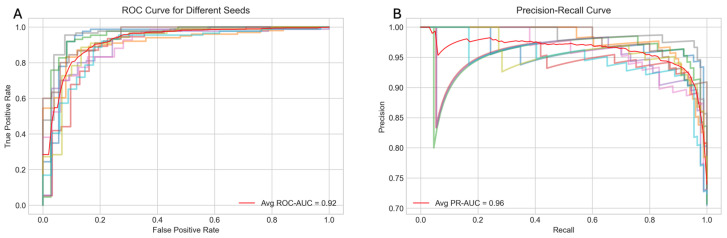
Panel (**A**) displays ROC curves generated from 10 different seeds, while Panel (**B**) shows the corresponding precision–recall curves across those 10 seeds.

**Figure 3:**
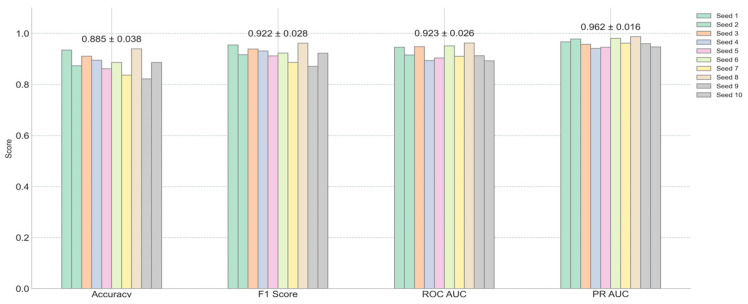
Performance evaluation (accuracy, F1 score, ROC AUC, and PR AUC) across multiple random seeds.

The performance of the model was evaluated using several metrics, including the receiver operating characteristic (ROC) curve, the precision–recall (PR) curve, accuracy, and the F1 score. Figure 2A displays the average ROC curve across the ten trials, achieving an area under the curve (AUC) of 0.92, indicating the strong discriminatory power of the model. Similarly, Figure 2B presents the average PR curve with a PR AUC of 0.96, demonstrating high precision and recall balance, which is particularly important for imbalanced healthcare datasets. Figure 3 offers a detailed view of the model’s performance across different random seeds for four key metrics: accuracy, F1 score, ROC AUC, and PR AUC. The average scores and their standard deviations, as annotated above each group, are as follows: accuracy is 0.885 ± 0.038, F1 score is 0.922 ± 0.028, ROC AUC is 0.923 ± 0.026, and PR AUC is 0.962 ± 0.016. The small standard deviations for ROC AUC and PR AUC suggest that the model’s performance is consistent and robust across different initializations.

Feature importance was extracted using SHAP (SHapley Additive exPlanations) to quantify the influence of each feature on the model’s predictions. SHAP values were computed by sampling 100 times from the test dataset, and the mean absolute SHAP value for the positive class (lung cancer) was calculated across all test samples. Among the 16 metabolites known to be associated with lung cancer, 4 of them, namely Choline, Betaine, Valine, and Fumaric Acid, were captured as part of the 30 most important features identified by the model. Abnormal Choline metabolism is a hallmark of malignant transformation, as it is essential for the synthesis of phosphatidylcholine, a key cell membrane component, and for cell signaling pathways that regulate proliferation and apoptosis. Elevated choline has been strongly linked to tumor aggressiveness and progression in lung cancer [22]. Fumaric Acid accumulates as oncometabolites and promotes metastasis, while Betaine supports aberrant Choline and methyl-donor metabolism in malignancy. Branched-chain amino acids such as Valine may also play a modulatory role in lung cancer lactate metabolism [23]. Age and height were among the 10 most important features.

To further assess the comparative performance of our M-GNN model against conventional machine learning approaches (Random Forest and Linear Support Vector Classifier). Even after balancing the training data with SMOTE, both tabular classifiers underperformed the M-GNN framework. Random Forest attained only 72.5% accuracy, 0.76 precision, 0.91 recall, 0.83 F1, and an AUROC of 0.56, while SVC yielded 71% accuracy, 0.75 precision, 0.91 recall, 0.82 F1, and an identical AUROC of 0.56. This persistent performance gap underscores the limitations of treating biomarkers as independent features, as compared to modeling their relations in a heterogeneous graph, which also accounts for hierarchical structures between pathways and diseases. Through its graph convolutional layers, M-GNN explicitly propagates information along metabolite–pathway–disease edges, embedding each patient’s biomarker profile within the broader biological context, thereby capturing multi-scale, mechanism-driven patterns of lung cancer metabolism that Random Forests and SVCs, which lack structural awareness, cannot learn.

Overall, the results demonstrate that the model achieves high performance across multiple metrics, with robust and consistent results across different random seeds. The training process shows effective learning, with some indications of overfitting that may warrant further regularization or early stopping strategies. The M-GNN methodology provides a comprehensive and integrative framework that effectively captures the intricate interplay between patient-specific metabolite expression, biological pathways, and disease associations. By constructing a heterogeneous graph enriched with HMDB-derived features and leveraging a multi-layer GraphSAGE architecture, the framework not only models fine-grained metabolic details but also contextualizes these within broader metabolomic networks. This robust multilayered approach underscores the potential of this approach to deepen our understanding of metabolic dysregulation in lung cancer and pave the way for enhanced precision in clinical diagnostics and targeted therapeutic strategies.

## 3. Discussion

**Figure 4:**
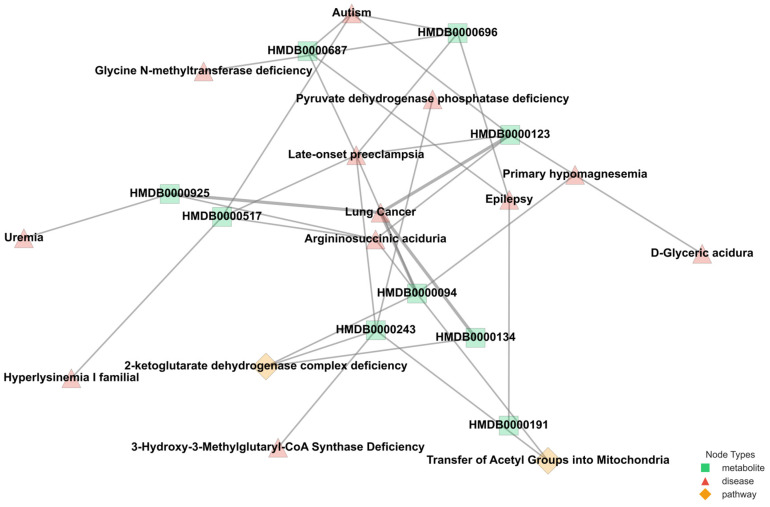
Metabolite–pathway–disease-sample subgraph visualizing a subset of a 3508-node, 114,415-edge heterogeneous graph, highlighting relational dependencies between metabolites, pathways, and diseases.

The varying connectivity patterns between node pairs play a crucial role in modeling metabolic interactions. Patient–metabolite connections follow a one-to-one structure, ensuring a direct mapping of metabolic activity, while metabolite–pathway and metabolite–disease relationships exhibit a one-to-many nature, reflecting the broader complexity of metabolic networks. The one-to-many relationships observed in metabolite–pathway and metabolite–disease connections highlight the intricate roles metabolites play in multiple biological processes. These associations, as annotated in the HMDB, emphasize the interconnected nature of metabolic pathways and disease states, which are critical for understanding disease mechanisms. By leveraging this structural diversity, M-GNN effectively captures both individual patient–metabolite interactions and the broader relational context of metabolic pathways and diseases. This dual-level representation enhances the model’s predictive accuracy. Figure 4 provides a representative illustration of the intricate connectivity within the metabolic network.

**Figure 5:**
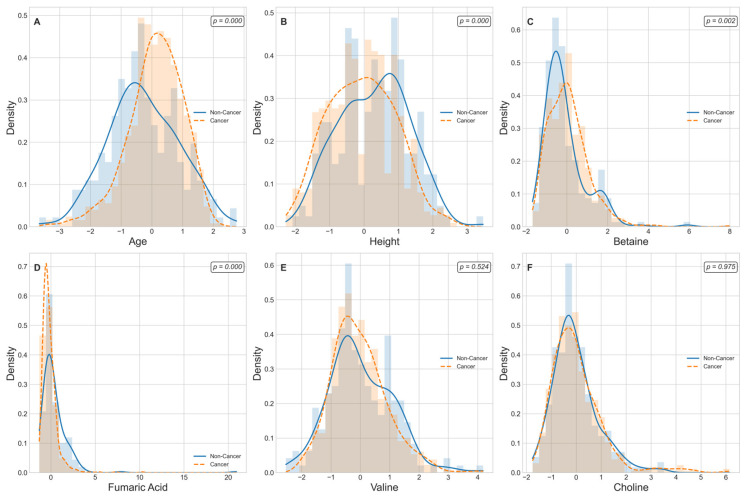
Kernel-density estimates of demographic and metabolite features stratified by lung cancer status. Cancer cases are represented in orange and control in blue. The six panels display the probability density functions (KDEs; solid lines) overlaid on histograms for (**A**) age, (**B**) height, (**C**) Betaine (HMDB0000043), (**D**) Fumaric Acid (HMDB0000134), (**E**) Valine (HMDB0000883), and (**F**) choline (HMDB0000097). Each panel reporting a two-sided Mann–Whitney U *p*-value with age, height, Betaine, and Fumaric Acid shows statistically significant differences (*p* < 0.01), whereas Valine and Choline distributions overlap (*p* > 0.05).

**Figure 6:**
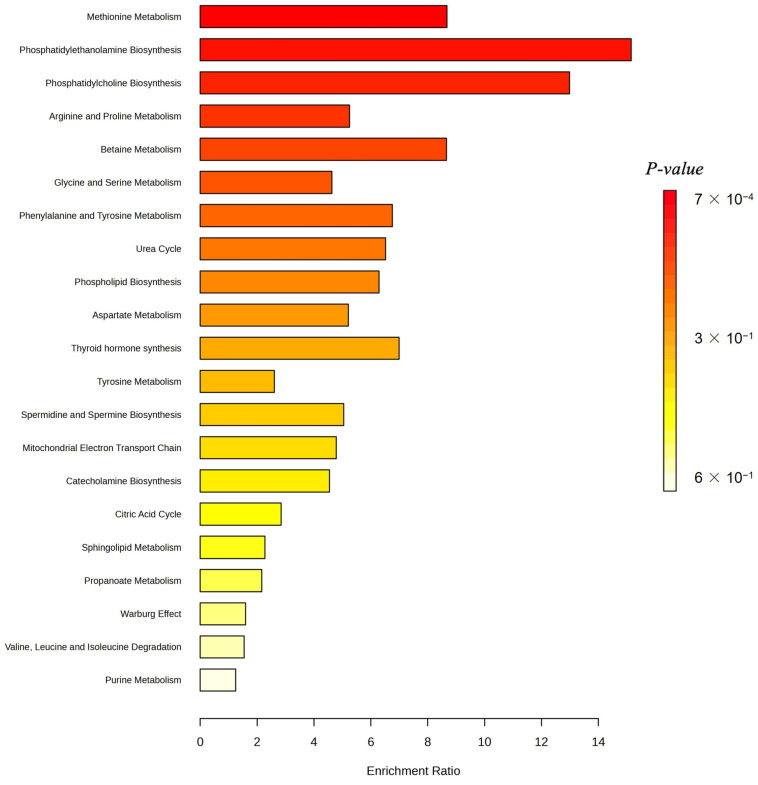
Metabolite-set enrichment of the top 30 M-GNN features using the MetaboAnalysis v.6.0 platform. Membrane–lipid biosynthesis pathways (phosphatidylethanolamine and phosphatidylcholine biosynthesis) and methyl-donor metabolism (methionine metabolism) emerge as the most enriched.

The visualization highlights how metabolites contribute to multiple pathways and diseases, reinforcing the need for models that can integrate such complex associations for improved disease prediction. The M-GNN model achieved a test accuracy of 89% (0.5 cutoff) and a ROC-AUC of 0.92, surpassing traditional tabular machine learning benchmarks, such as the 83% accuracy reported using conventional methods on similar metabolomics datasets [8]. These results, visualized in Figure 5 and Figure 6, demonstrate rapid convergence in less than 400 epochs and robust discriminative power, particularly for early-stage lung cancer cases (70% Stages I–II), aligning with the metabolomics-driven early detection paradigm [24,25,26,27]. The three most frequently observed pathways linked to the most influential metabolites, namely, dimethylglycine dehydrogenase deficiency, glycine–serine–threonine metabolism, and transcription/translation, are implicated in a diverse array of diseases. Conditions ranging from metabolic syndromes (e.g., diabetes mellitus type 2, obesity) to multiple cancers (e.g., pancreatic, colorectal) demonstrate established connections with these pathways. In lung cancer specifically, the hijacking of one-carbon and amino acid metabolism (particularly glycine, serine, and threonine) fosters accelerated tumor growth, augmented nucleotide production, and balanced redox homeostasis [28]. Moreover, inflammation-driven disorders such as rheumatoid arthritis, ulcerative colitis, and Crohn’s disease share pro-inflammatory and transcriptional dysregulation mechanisms with malignancies, thereby generating an environment conducive to cancer progression [29,30].

**Figure 7:**
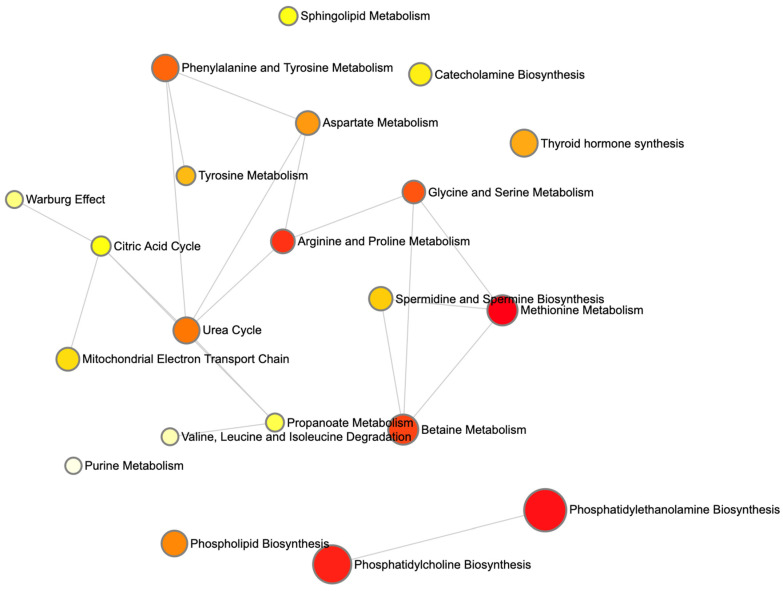
Network overview of the top 30 M-GNN features using the MetaboAnalysis 6.0 platform. Nodes represent enriched pathways sized based on enrichment magnitude and colored based on significance, with darker colors having a larger *p*-value. Edges connect pathways that share one or more of the top 30 metabolites. Distinct clusters, such as lipid-biosynthesis versus one-carbon/amino acid modules, highlight how key metabolic routes interlink via shared biomarkers.

Figure 5 displays kernel-density estimates (KDEs) for age, height, and four lung-cancer-associated metabolite levels that are ranked among the top 30 predictive features grouped by cancer status. A Mann–Whitney U test reveals that age and height exhibit highly significant distributional differences between non-cancer and cancer cohorts (*p* < 0.001 for each), consistent with known epidemiological risk factors. Among the metabolic markers, Betaine and Fumaric Acid demonstrate pronounced shifts in density curves, with cancer cases showing markedly higher Betaine levels (*p* = 0.002) and lower Fumaric Acid levels (*p* = 1.6 × 10^−10^) relative to controls. In contrast, Valine and choline yield overlapping KDEs and fail to achieve statistical significance (*p* > 0.05), indicating comparable plasma concentrations across groups. These findings both confirm the robust dysregulation of specific metabolites in lung cancer and support the selective inclusion of significant edge-weight features in the M-GNN model’s graph representation, while excluding non-discriminatory biomarkers. We further performed pathway enrichment analysis to identify which biological routes are disproportionately represented among the most important features. Figure 6 displays the enrichment ratios for all metabolic pathways significantly over-represented among the 30 most predictive metabolites identified by our M-GNN model. Phosphatidylethanolamine biosynthesis, phosphatidylcholine biosynthesis, and methionine metabolism rank among the highest, indicating that membrane lipid remodeling and methyl-donor pathways are disproportionately represented in our top features. Figure 7 renders these enriched pathways as nodes in a network, with node size scaled based on the enrichment ratio and node color again reflecting statistical significance. Edges link pathways that share one or more of the top 30 metabolites. The graph reveals a cluster of lipid-biosynthesis pathways (PE and PC) and another cluster around amino-acid and one-carbon metabolism (methionine; glycine and serine; and Betaine).

**Figure 8:**
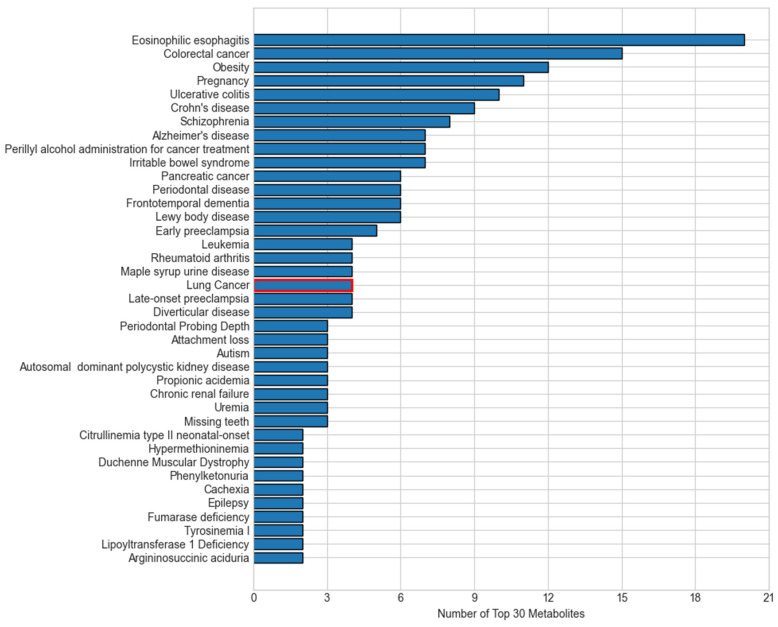
Number of top 30 metabolites associated with at least 2 diseases, illustrating meaningful overlap between M-GNN top metabolites and diseases that share similar pathways with lung cancer. Lung cancer, shown in red at the center of the chart, is linked to the 4 of the top 30 M-GNN features.

Many of the top diseases linked to our top 30 metabolites converge on the same biological processes that drive lung carcinogenesis, explaining their overlap (Figure 8). First, chronic inflammatory and autoimmune conditions such as eosinophilic esophagitis, ulcerative colitis, and Crohn’s disease reflect persistent immune activation and cytokine release, which create a pro-tumorigenic microenvironment that can also promote lung tumor initiation and progression [31]. Metabolic disorders like obesity induce insulin resistance and altered adipokine signaling, fostering cell proliferation and resistance to apoptosis in pulmonary tissue [32]. Other cancers, such as colorectal cancer, pancreatic cancer, and leukemia, might share environmental exposures such as smoking, DNA repair deficiencies, and similar shifts in amino acid and lipid metabolism with lung tumors [33]. Neuropsychiatric and neurodegenerative diseases, including schizophrenia, Alzheimer’s, and frontotemporal dementia, increasingly show evidence of mitochondrial dysfunction and oxidative stress, which are also hallmarks of cancer cell bioenergetics [34]. These disease–metabolite links demonstrate that the key M-GNN biomarkers capture the chronic inflammation, metabolic reprogramming, and redox imbalance pathways characteristic of lung cancer.

Despite these strengths, several limitations warrant consideration. First, the computational complexity of graph-based methods poses scalability challenges. With 3508 nodes and 114,415 edges, processing times increase significantly with larger cohorts, limiting clinical deployment feasibility. Optimizing with attention mechanisms, such as those in GAT layers, or pruning non-essential edges could mitigate this issue. Second, the model’s focus on 107 metabolites would benefit from enhanced feature selection to manage dimensionality. The M-GNN model results demonstrate that incorporating metabolomics data into a GNN-based framework significantly refines lung cancer detection and prognosis, aligning with the broader trend of using graph architectures for complex biomedical challenges [35]. While prior imaging-based GNN studies have excelled in survival analysis and early-stage detection using CT scans, our multi-omics approach underscores the value of integrating metabolite profiles and clinical factors to capture the metabolic intricacies of tumor biology. Moreover, such fusion strategies can be extended to genomic and transcriptomic data, as recently shown in dynamic adaptive deep fusion networks [36], potentially improving predictive accuracy and uncovering novel therapeutic targets. Taken together, these findings illustrate how GNN methodologies can bridge the gap between diverse data modalities, enabling precise oncology solutions that are both highly accurate and biologically interpretable.

## 4. Materials and Methods

The metabolite graph neural network (M-GNN) introduced in this paper constructs a heterogeneous graph that integrates metabolomics and demographic data with biological pathways and diseases. To explore the relationships between pathways, diseases, and metabolites, we analyzed 107 metabolites in our dataset that either have established normal ranges in the Human Metabolome Database or are associated with lung cancer within the HMDB. Subsequently, we extracted all pathways involving these metabolites and identified diseases known to be associated with them, as documented in the HMDB. Additionally, we enriched the metabolite nodes with HMDB-derived normal adult ranges, including lower limit, upper limit, and average expression levels. Patient features were systematically categorized into two groups: demographic variables—encompassing attributes such as gender, race, smoking status, smoking current, smoking past, age, height, weight, BMI, and cigarette packs per year—and metabolite measurements associated with the 107 metabolites. To ensure consistency, numerical features were normalized to a [0,1] scale using the StandardScaler, with missing values imputed based on K-Nearest Neighbors (KNN). The KNN imputation method estimated missing values based on the two nearest neighbors, applying a uniform weighting scheme.

To enrich each patient’s metabolic profile with established biochemical knowledge, we constructed a single heterogeneous graph by fusing three curated bipartite relationships. First, we linked every metabolite node to the pathways in which it participates by adding the “involved in” edges of unit weight. Second, we connected metabolites to diseases via “associated with” edges. Finally, we encoded patient-specific metabolite expression levels and attached these values as a “has concentration” edge weight. Each edge also carried a one-hot relation identifier so that the GNN learns distinct message-passing rules for pathway membership, disease association, and concentration abnormality. By bringing together metabolite-specific pathway structure, literature-curated disease links, and patient-level biomarkers, our M-GNN leverages both qualitative context and quantitative perturbation to improve classification while maintaining clear biological interpretability.

A heterogeneous graph, G = (V, E), was constructed using NetworkX to model relational dependencies between patients, metabolites, diseases, and pathways, drawing inspiration from graph-based biological modeling. Patient nodes contained 10 demographic features and 107 metabolite expression levels, while metabolite nodes utilized the 3 HMDB normal range features. Disease and pathway node features were set to [0]. Patients were linked to metabolites via weighted edges defined as metabolite expression levels, with edge type defined as has concentration. Metabolites were connected to diseases (weight 1.0, relation = associated with) and pathways (weight 1.0, relation = involved in). Patient node labels were set to [0,1], while all other node labels were defined as [−1]. Patients with a history of smoking were linked to the lung cancer disease node, with a weight of 0.8 and a relation type of risk_factor, to reflect increased risk. For the 16 metabolites known to be associated with lung cancer, the corresponding edge weight was set to 2. The graph was converted into a PyTorch Geometric 2.7.0 Data object, encoding node features x ∈ R|V| × 117, labels y ∈ R|V|, symmetrized edge indices, and weights. The M-GNN model integrates edge weights into its graph convolutional layers through an adjacency matrix module, followed by a sequence of convolutional and dense operations. Edge weights were transformed using a sigmoid function and scaled by a learnable parameter, σ, initialized at 0.5, which the model adjusts during training to fine-tune their influence.

The first convolution layer, defined as a SAGEConv, begins with a standard unweighted mean aggregation of neighbor features. Subsequently, edge weights are applied by scaling the features of source nodes with their corresponding edge weights and normalizing by each target node’s degree to replicate SAGEConv’s mean aggregation logic. This weighted result is then blended with the original unweighted aggregation using a 50-50 average (Equation (1)), ensuring that edge weights augment the aggregation without overshadowing the original node feature signals.

(1) $$\begin{array}{rr} x_{1} & =\frac{\left(x_{1}+A_{\mathrm{weighted}}\right)/d}{2} \end{array}$$

The weighted adjacency matrix, $A_{\mathrm{weighted}}$, incorporates a learnable scaling parameter, σ, that modulates the edge weights before multiplying them with the corresponding node features. Mathematically, this is expressed as

$$A_{\mathrm{weighted}}=\left(\sigma \cdot w_{0}\right)\cdot x_{1}\left[e_{\mathrm{src}}\right]$$

where the source node weights, $w_{0}$, extract edge weights, $e_{0..n}$, for source nodes, $V_{0}$, based on their connectivity to target nodes, $e_{\mathrm{tgt}}$. Initially, $A_{\mathrm{weighted}}$ is set to a constant value of 0.5. It is subsequently updated by incorporating the weighted contributions of source node features, $x_{1}\left[e_{\mathrm{src}}\right]$, scaled by $w_{0}$. The degree of each node, d, is computed as

$$d=\max\left(\underset{i}{\sum }1_{t_{i}},1\right)$$

This ensures a minimum degree of 1. Finally, the updated node features, $x_{1}$, are normalized and averaged, incorporating the effects of the adjacency matrix and node connectivity.

**Figure 9:**
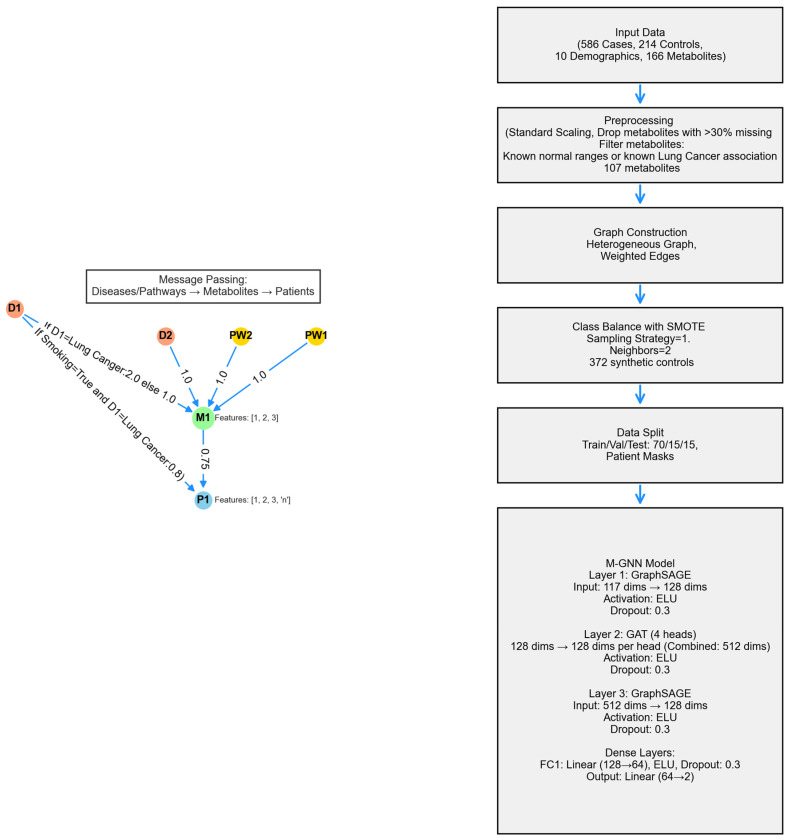
Architecture of the M-GNN model, depicting the GraphSAGE layers aimed at learning cancer status based on metabolite expression levels and their known disease and pathway associations.

The model’s second layer is a GATConv layer, where scalar edge weights (edge_weight) are incorporated as edge attributes, influencing attention coefficients and allowing the model to dynamically prioritize connections. This layer outputs a 512-dimensional feature representation by concatenating 128 channels from four attention heads. Batch normalization is then applied, followed by an ELU activation and dropout (0.3) for regularization. Next, a SAGEConv layer further processes the features, reducing the dimensionality to 128. A weighted adjacency adjustment is performed, where contributions from source nodes are aggregated and normalized using the degree of target nodes, as described in the initial SAGEConv layer above. This adjustment balances feature propagation and ensures stability in the learned representations. The processed features undergo batch normalization, ELU activation, and dropout (0.3). The final stage consists of two fully connected layers: the first reduces feature dimensionality from 128 to 64, applying ELU activation and dropout (0.3), while the second generates the final logits for two-class classification. A diagram of the model architecture is shown in Figure 9.

Training was conducted using the AdamW PyTorch 2.7 optimizer , configured with a learning rate of $5\times 10^{-4}$ and a weight decay of $1\times 10^{-4}$. To dynamically adjust the learning rate, a ReduceLROnPlateau scheduler was employed, reducing the learning rate by a factor of 0.5 if validation loss did not improve for 300 consecutive epochs, with a minimum learning rate threshold of $1\times 10^{-6}$. To handle the remaining class imbalance in the dataset after SMOTE, a weighted cross-entropy loss function was utilized. Class weights were computed based on the inverse frequency of class occurrences in the training labels, normalizing them to sum up to one. Additionally, label smoothing (0.1) was applied to prevent overconfidence in predictions and improve generalization. The model was trained for up to 1500 epochs, with an early stopping mechanism implemented if the validation F1 score did not improve over 300 consecutive epochs. Evaluation was performed on a held-out test set using multiple performance metrics, including accuracy, precision, recall, F1 score, and area under the receiver operating characteristic curve (AUROC), to provide a comprehensive assessment of classification performance. To ensure reproducibility, the 10 random seeds were fixed across all stages of training and evaluation.

The heterogeneous graph is composed of 107 metabolite nodes, 231 disease nodes, and 2014 pathway nodes—totaling 3508 nodes connected by 114,415 edges. Most connections (206,572 edges) reflect the expression levels of metabolites from 800 actual participants and 196 simulated controls, while 5873 edges link pathways to metabolites. Table 1 provides a summary of the graph.

## 5. Conclusions

This study introduces M-GNN, a graph neural network framework leveraging GraphSAGE, designed for early lung cancer detection using a heterogeneous graph integrating metabolomics and demographic data from 800 plasma samples (586 cases, 214 controls), enriched with Human Metabolome Database (HMDB) annotations. The model achieved a test accuracy of 89% and an ROC-AUC of 0.92, converging within 400 epochs and exhibiting consistent performance across ten random seeds. The model effectively captures complex metabolic interactions, identifying key biomarkers like choline, Betaine, Valine, and Fumaric Acid, highlighting height and age as dominant risk factors. Despite its strengths, limitations include potential biases from synthetic data and the computational demands of graph-based methods, suggesting future refinements with attention mechanisms or real-world datasets. M-GNN advances precision oncology by offering a scalable, interpretable tool for lung cancer screening, with the potential to enhance survival rates through early detection and personalized treatment strategies. Future work should focus on validating the framework with clinical cohorts and optimizing computational efficiency to broaden its applicability in metabolomics-driven diagnostics.

## Figures and Tables

**Table 1:** Graph statistics summary.

Metric	Value
Total Number of Nodes	3508
Total Number of Edges	114,415
Synthetic Nodes Generated	196
Node Type Counts	
Pathways	2174
Metabolites	107
Diseases	231
Samples (Lung Cancer and Control)	996
Edge Type Counts	
Metabolite–Pathway	5873
Metabolite–Patient	106,572
Metabolite–Disease	1247
Smoking–Lung Cancer	723

## Data Availability

The data are unavailable due to privacy or ethical restrictions. The code has been made available on GitHub at https://github.com/miliana/M_GNN (accessed on 2 May 2025).
